# Digital Health at Enterprise Scale: Evaluation Framework for Selecting Patient-Facing Software in a Digital-First Health System

**DOI:** 10.2196/43009

**Published:** 2023-04-07

**Authors:** Martin Shapiro, Sondra Renly, Ali Maiorano, Jerry Young, Eli Medina, Aaron Neinstein, Anobel Y Odisho

**Affiliations:** 1 Department of Family and Community Medicine, University of California, San Francisco San Francisco, CA United States; 2 Center for Digital Health Innovation, University of California, San Francisco San Francisco, CA United States; 3 Department of Medicine, University of California, San Francisco San Francisco, CA United States

**Keywords:** artificial intelligence, digital health pathway, eHealth, enterprise digital health, evaluation framework, framework, healthcare delivery, healthcare system, intelligent care, intelligent system, privacy, security, service delivery, systems design, telehealth, telemedicine

## Abstract

The digital transformation of our health care system will require not only digitization of existing tools but also a redesign of our care delivery system and collaboration with digital partners. Traditional patient journeys are reactive to symptom presentation and delayed by health care system–centric scheduling, leading to poor experience and avoidable adverse outcomes. Patient journeys will be reimagined to a digital health pathway that seamlessly integrates various care experiences from telemedicine, remote monitoring, to in-person clinic visits. Through centering the care delivery around the patients, they can have more delightful experiences and enjoy the quality of standardized condition pathways and outcomes. To design and implement digital health pathways at scale, enterprise health care systems need to develop capabilities and partnerships in human-centered design, operational workflow, clinical content management, communication channels and mechanisms, reporting and analytics, standards-based integration, security and data management, and scalability. Using a human-centered design methodology, care pathways will be built upon an understanding of the unmet needs of the patients to have a more enjoyable experience of care with improved clinical outcomes. To power this digital care pathway, enterprises will choose to build or partner for clinical content management to operationalize up-to-date, best-in-class pathways. With this clinical engine, this digital solution will engage with patients through multimodal communication modalities, including written, audio, photo, or video, throughout the patient journey. Leadership teams will review reporting and analytics functions to track that the digital care pathways will be iterated to improve patient experience, clinical metrics, and operational efficiency. On the backend, standards-based integration will allow this system to be built in conjunction with the electronic medical record and other data systems to provide safe and efficient use of the digital care solution. For protecting patient information and compliance, a security and data management strategy is critical to derisking breeches and preserving privacy. Finally, a framework of technical scalability will allow digital care pathways to proliferate throughout the enterprise and support the entire patient population. This framework empowers enterprise health care systems to avoid collecting a fragmented series of one-off solutions but develop a sustainable concerted roadmap to the future of proactive intelligent patient care.

## Introduction

The health care system in the United States is expensive, inefficient, inequitable, and delivered through a fragmented patient experience. The United States spends almost 1 of every 5 dollars on health care, and US $32 billion of health care expenditures are on avoidable emergency department visits and admissions with operational inefficiencies [1-4]. In many industries, the process of upgrading the business with technology, also known as digital transformation, has catalyzed dramatic improvements in efficiency, consumer experience, and cost [5]. By placing patients at the center of their care and delivering a seamless, data-driven care experience, well-designed and implemented digital health tools can improve care quality while also delivering value.

Health care’s first attempt at entering the age of a digital user experience was to replace centuries-old paper records with electronic health record (EHR) systems, but this did not initially change care delivery. The paper record was simply displayed on screen, with minimal integration, no intelligence, and no clinical decision support. While these systems have improved dramatically, the focus has remained on the provider as the primary EHR user delivering care in iteratively updated workflows. With the rise of consumerism in health care and increased emphasis on patient engagement, the next generation of digital health innovation will move beyond creating a digital copy of the legacy care delivery system to a reimagination of technology-enabled patient-centered care [6-8].

Large enterprise systems have been catalyzed by the COVID-19 pandemic to transform broader disease management. Synchronous, real-time, in-person care delivered by physicians is episodic, inefficient, infrequent, and expensive. It misses the totality of a patient’s lived health experience and may not be the best first point of contact for all clinical conditions. Digital health pathways, on the other hand, provide an opportunity to improve health outcomes by enabling the delivery of continuous, proactive, and personalized care [9]. To enable this digital transformation, health care systems must reimagine the care delivery model and identify whether they need to buy, build, or partner to acquire the capabilities necessary to deliver this care.

We aim to provide an evaluation framework for the capabilities required in a digital health platform to enable enterprise health care systems to deploy remote care at scale across various care conditions, such as primary care, complex chronic care, periprocedural care, and inpatient care.

## What Is a Digital Health Pathway?

A digital health pathway combines an automated patient engagement platform with clinical workflows to seamlessly navigate a patient through various care experiences, such as telemedicine visits, digital check-ins, in-person visits, remote monitoring, patient-reported outcomes assessment, and appropriate escalations to the care team.

**Figure 1:**
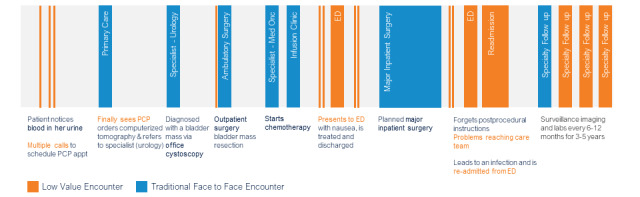
Current patient bladder cancer journey. ED: emergency department; PCP: primary care provider.

Existing care delivery pathways reflect the reactive, episodic, and nonpersonalized care workflows across ambulatory, periprocedural, and inpatient care settings. In the existing care model, staff lack the time and tools to provide a personalized, patient-centered experience of timely access to primary care and specialist physicians, assessment for complications, education, and surveillance. For example, let us walk through a journey of current patients with bladder cancer (Figure 1): they may have noticed symptoms of blood in their urine leading to a visit with their primary care provider leading to urine tests, referral to urology specialist, diagnosis by office cystoscopy, surgery, chemotherapy, and surveillance, which were complicated by delays of care and avoidable adverse events. We find this journey reactive, episodic, inefficient, and nonpersonalized. At each step of scheduling, referrals, and procedures, the patients can experience delays in care, symptom exacerbation, or complications. Most systems are instrumented to react and respond to patient outreach, instead of proactive monitoring and management.

**Figure 2:**
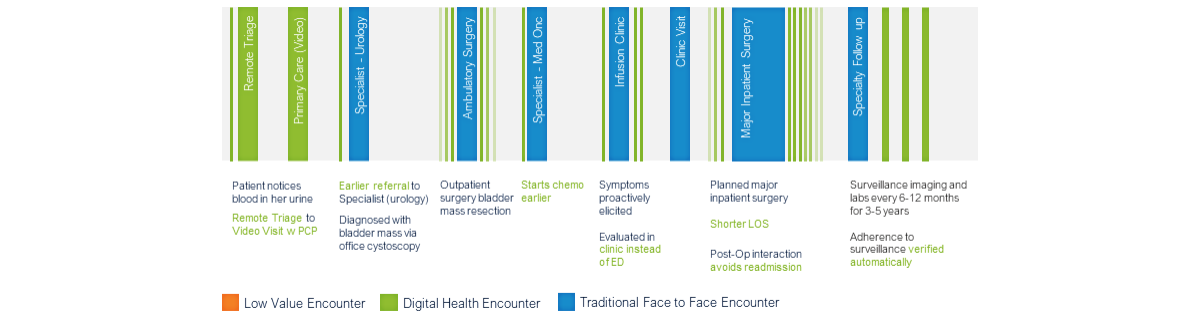
Bladder cancer digital health pathway. ED: emergency department; LOS: length of stay; PCP: primary care provider.

The future state of the bladder cancer digital care pathway (Figure 2) is a proactive, fluid, efficient, and personalized journey. This digital pathway strings together the key critical anchor events of referral, bladder mass diagnosis and resection, and chemotherapy through digital augmentation of patient education, logistical planning, symptom assessment, and surveillance adherence. Targeting the frictions of delay in diagnosis, adverse events, and missed surveillance, this care pathway uses different digital tools such as messaging, data collection, and health system integration to streamline the journey of patients with bladder cancer. Remote monitoring and symptom assessments identify patients who have a worsening condition that requires a clinical escalation, reducing unplanned emergency room visits, or admissions [10]. For patients with thoracic cancer, who completed telemedicine visits were associated with reduced emergency department visits and hospital admissions [11]. The digital care pathway is a paradigm shift from our health care system as it currently exists, which is reactionary to patient’s worsening symptoms and prone to a Swiss cheese model of errors. Rather than relying on individuals of patients, providers, and staff members, an intelligent digital pathway unlocks the opportunities of what should be done rather than what can be done with the constrained resources of time (eg, phone reach out and panel management) and clinic capacity (eg, limited number of patient rooms and provider appointments). For example, in colorectal cancer surveillance, a multimodal engagement of electronic message, telephone, and letter significantly improved surveillance [12]. In a systematic review, SMS text messaging improved patient satisfaction with cancer care and supported medication adherence [13]. Only with a digital care pathway can proactive and personalized monitoring be delivered at scale.

## Framework for the Remote Care Program Development

Although the patient engagement capabilities in the dominant EHRs are improving, many enterprises will find themselves turning to third-party solutions to facilitate a modern, innovative digital patient experience. Given the landscape of potential vendors to enable these digital experiences, an enterprise must identify and agree on the selection criteria for a suitable technological partner. The stakeholders represent users involved including providers, staff, patients, and the operational, financial, and strategic leadership across clinics, laboratories, imaging centers, and inpatient facilities [14]. Understanding the needs of patients and staff through current and future journeys and workflow mapping to obtain buy-in is critical for the successful implementation of any program. In addition, leadership will assess the investment needed for the solution and metrics for success, including return on investment and strategic value.

To guide stakeholders through selection of a digital development partner, various evaluation frameworks are used to characterize the organization and their products’ ability to service clinical needs at scale. In the American Medical Association’s Digital Health Implementation Playbook, their best practice of vendor selection is categorized across business, information technology, security, usability, customer service, and clinical validation [15]. This evaluation methodology filters partners through their ability to meet the needs of the clinical use case, achieve return on investment, and derisk with security and IT integration.

We propose the following set of criteria ranging from design to technical requirements that focus on the capabilities needed for an enterprise health care system to implement digital health pathways at scale: human-centered design, operational workflow, clinical content management, communication channels and mechanisms, reporting and analytics, standards-based integration, security and data management, and scalability.

## Human-Centered Design

Human-centered design is the creative approach to problem-solving that directly engages with the people for whom the design is intended [16]. This design’s mission and methodology is core to defining the development of patient-centered digital care pathways. A key tool is developing experience principles that help align teams around core experiences and guide them to make decisions around the holistic experience, rather than specific touch points. Patient-centric care experience principles are grounded in empowering patients to drive their own care, have the best clinical outcomes possible, and experience a frictionless care journey, minimizing the burden of care.

For example, an experience principle of “empowering patients” comprises developing health literacy education, including signs of exacerbations or complications, increasing asynchronous and synchronous access to care, and owning health care data. This experience principle also extends to proxies for patients, such as caretakers for children or elderly and conservators.

Another experience principle is “equity and access across the community,” specifically for language and reading level. In an analysis of patient education materials provided by a major vendor, reading levels were found to be at high school or college reading levels, while the average person in the United States reads at or below 8th-grade level [17,18].

Starting with a detailed service blueprint creates a human-centered representation of the relationships among different service components—people, props (physical or digital), and processes. This tool visualizes organizational processes in order to facilitate services that provide useful, desirable, and effective user experiences [19]. To better understand the needs of patients, providers, and staff, one can step into their shoes through interviews, observations, and participatory design sessions. Teams synthesize their learnings into care journey maps that highlight the challenges and barriers to high-quality care. In addition, engagement with Patient and Family Advisory Councils, which are standing advisory committees of patients and family members, can also serve as a meaningful way to engage with care needs to better design pathways [20,21]. After understanding the challenges that patients, providers, and clinical staff face in improving care, the enterprise should align on a set of experience principles [22,23].

In developing a novel digital care pathway, we want to ensure that we are engaging our clinical experts and patients to curate journeys that are supported by evidence through timely assessments, interventions, and surveillance to improve clinical outcomes. Lastly, the experience must be frictionless with minimal cognitive, time, and human resource burden for providers and staff to deploy and engage the digital assets while facilitating an intuitive interface for patients and their families. This intuitive interface for patients describes the way in which patients will be able to interact with the health care system, such as SMS text message, email, phone calls, video visits, symptom questionnaires, or photo uploads.

## Operational Workflow

Digital tools are only as effective as their integration with the people and processes that work with them. Poor integration of digital tools can lead to lack of user adoption and personnel burnout. Operational workflows for digital tools need to have frictionless user experiences for the end user (patient, nurse, physician, etc) and streamlined integration into the clinical processes.

Ensuring that the digital tool provides an efficient user experience will ease its integration into the clinical workflow. For example, a patient with bladder cancer completes their postoperative symptom surveys to assess for signs of infection, how is that information presented to and acted upon by the care team? An example of a poor clinical workflow integration would be to force a care team member to sign into a separate vendor dashboard, manually review patient responses, switch to the EHR for additional context, contact the patient, and place orders. A Substitutable Medical Applications, Reusable Technologies on Fast Healthcare Interoperability Resource integration may allow the vendor tool to launch within the EHR with appropriate clinical context, but critical data are not written back into the EHR, meaning they cannot be easily used for additional alerts or documentation. Ideally, concerning results would be automatically escalated into the care team’s EHR in-basket, and relevant data would be written into the EHR directly. This allows use of preexisting clinical workflow (eg, the EHR inbox) with enough clinical context for providers to triage findings and enable next steps, such as placement of orders, patient outreach, and scheduling of telehealth visits. Working with clinical operational leadership and an interprofessional working group to ensure buy-in and willingness to invest in staff resources is critical to the ultimate success of a digital care pathway.

## Clinical Content Management

Content creation, which includes patient education materials and clinical decision algorithms, is expensive and time-consuming, requiring content management system, clinical experts, and governance to keep it up-to-date and relevant. While much patient education content exists and is licensable, each enterprise and service line will inevitably need to customize this to their care standards and workflows. An enterprise should decide how much of this content creation they would like to own and if they plan to drive development. Important differentiation aspects of clinical creation include the ability for the health enterprise to customize content using a friendly, nontechnical interface, guidance from vendor clinical support staff, and speed to develop or modify care pathways. It is also important to define who owns any intellectual property created during pathway development at the start of the vendor relationship.

Vendors, depending on their maturity and scope, have a varying depth and breadth of clinical content. After an initial review to confirm if the existing content matches enterprise needs, close scrutiny from internal subject matter experts is required to confirm the quality and usability of content. Areas of clinical content can range from chronic disease management, procedures, to acute symptom management. For each condition or care pathway, one can evaluate the depth of the clinical assets that may include patient-reported outcomes (symptoms), disease-specific education, remote monitoring integration, disease journey touch points, patient and provider dashboards, and support for multiple languages. It is critical to determine if the clinical content has been validated (data from peer-reviewed studies or trusted sources), was it created by the vendor, licensed, or repurposed some other source.

Optimally, an enterprise needs best-in-class clinical care pathways, which can be kickstarted with an existing library of content with frictionless customization and iteration. Key features are the availability of customizable white-labeled content, easy pathway modification, the ability to build content without technical support, and ownership of new intellectual property.

## Communication Channels and Mechanisms

The digital care pathway presents an opportunity to better meet a patient where they are using digital infrastructure. To place the patients at the center of their care, it is paramount to empower them with tools to communicate their questions and the state of their health. For outgoing interactions sent to the patient, communications can be written, digital text, audio, photo, or video. As heath care systems move beyond hard copies of patient education materials, they must take a comprehensive approach to digital delivery, which can include EHR portal, secure messages, email, SMS text messages, web content, videos, public social media engagement, and personalized chatbots. Audio communication can range from live human-initiated phone calls to automated experiences, including robocalls and interactive voice response calls. For visual communications, multimedia messages of photos and videos, such as educational materials or video chatting, can expand the scope of web-based clinical offerings.

In addition to outbound communication to the patient, incoming communications from the patient include survey responses of patient-reported outcomes, multimedia such as photos and videos, and data from medical devices, either medical or consumer-grade. Surveys capturing patient’s symptoms, quality of life, and other qualitative assessments can be critical in providing assessments of the patient in between clinic visits, leading to better symptom management, clinical outcomes, patient engagement, and resource uses [21]. Features enabling bidirectional photo transmission and sending patient video allow the care pathway to better triage, escalation of care, or expedite referrals, including photo referrals for malignancy workup in dermatology [24]. Integration with devices such as blood pressure cuffs and pulse oximeters complement the symptom assessments with quantitative measurements that can further support intelligent clinical care pathways.

Importantly, the digital patient engagement platform must enable the secure transmission, integration, and management of patient device data with the EHR.

## Reporting and Analytics

A digital health intervention generates significant amounts of not only clinical data but detailed logs outlining patient use and engagement. Frequent reporting is critical to assess any clinical and operational changes, but also how effectively the patients are able to use the tool and their continued engagement.

Engagement metrics are usually obtained from the vendor and focus on factors such as enrollment, engagement, completion of modules, and volumes of messages transacted. Tracking of these metrics can be an early measure of program success and will be the first to vary as changes are made to the program. The definitions of engagement can vary wildly, and this is an area where few clinicians have experience with analysis and interpretation of results. Clinical metrics will differ for each disease state and use case, with examples including laboratory value targets, vital sign goals, and clinical screenings [25]. These can be measures of treatment adherence, treatment response, avoidance of complications, emergency room visits and readmissions, and long-term outcomes. Operational metrics focus on the impact a digital health tool has on care delivery, such as numbers of secure messages or phone calls, staff time spent, appointment cancellations, and improved time to care. Importantly, while the vendor can usually provide engagement metrics, the health system analytics team must integrate these data with clinical data to obtain more meaningful and actionable insights. The enterprise analytics team must also be resourced to support development of routine reporting and dashboards, as vendor dashboards are usually quite narrow and cannot reflect the complexities of each organizations data infrastructure and reporting needs. This can be a significant investment but is critical to evaluating pathway success and iterating to improve the patient experience.

In assessing a vendor’s readiness to support reporting and analytics, the team must evaluate how much the vendor provides “out of the box,” how customizable their dashboards are, and do they provide access and support to export raw data for more detailed analytics. Fundamentally, health enterprises must determine the time and investment it will take to setup and maintain an actionable analytics system for the needs of the digital care pathway.

## Standards-Based Integration

Technical integration is critical to ensure the solution provides safe and efficient integration with the existing care delivery processes in a way that is empathic, personalized, and modern, as well as being compliant, secure, and scalable. On an industry level, most integrations have been to facilitate hospital-to-hospital communication rather than directly supporting clinical decision-making [26]. However, in a digital care pathway, integrations of clinically relevant information ensure visibility to all care team members, decrease the cognitive burden, and reduce errors across all users in manually inputting information or transferring them across platforms.

The critical integrations will be across systems of the enterprise health care system, the patient, and supporting partners within the digital care pathway. The decision of the amount of integration is a balance between the functionality benefits of the integration and the investment to deploy the integration in terms of cost and time.

When having this integration goal, one can evaluate possible vendors for their ability to carry out this integration. To evaluate vendors, assess what their stated integration abilities are, case studies exemplifying prior integration abilities, the maturity of their capabilities, technical capacity, and use of industry standards. Use of industry standards integration such as fast health care interoperability resources or Health Level 7 will decrease technical burden for the medical enterprise team to incorporate the vendor [27]. Based on the vendor’s capabilities and the needs of the digital tool, a product roadmap can be created from the initial minimal viable product to a future vision of the solution. The minimal viable product would include the least amount of integration possible to allow this product to function and provide the value needed to demonstrate the solution is possible. In the long-term vision, further integrations can be explored, ranging from EHR to medications, devices, transportation, and other third parties.

One of the main integrations with the health care system will be with the EHR. At a high level, integrations with an electronic medical record (EMR) can be classified as read or write. A read-only integration will allow a third party to have access to view select data within the system of record. These data can either be displayed or even used as inputs into a third-party tool. However, a read-only integration will now allow the EMR to receive new information or have data changed by a third-party tool. A simple write function allows a tool to upload files such as PDFs into the record, but a sophisticated write functionality empowers a tool to edit, change, or add information into the system of record. A write capability allows for all of the new data information to be up to date in the EMR, which allows clinicians to access the data without needing to be enrolled in a third-party application. Furthermore, incorporation of the data within the EMR ensures that data generated by the third party will remain accessible if the app goes out of business, contract ends, or some other dissolution of the partnership occurs. Within read or write, the levels of integration will be specific to the needs of the proposed solution and may include, mobile app, provider context, EMR inbox, scheduling, etc. Legally, read or write will present its own considerations for the terms of the contracting of data ownership, privacy, and compliance. This integration may be facilitated directly by the vendor or in collaboration with integration technology partners.

Clinical data streams, however, do reside only in the EMR. Integrating additional data may have distinct challenges, such as meds being managed and updated in multiple different systems (by providers not within your primary system), remote patient monitoring data including blood pressure cuffs, weight scales, and other biometrics. These integrations can be facilitated directly with device vendors such as iHealth or with data aggregators including Apple HealthKit and Google Fit.

Other third parties that interface with patients include transportation, laboratories, and payment. As enterprise health systems modernize their nonemergent transportation options, integration with ride shares such as Lyft and Uber can help remove the frictions of patients making it to their appointments. Laboratory integrations will allow patients to effortlessly coordinate necessary blood work and understand their results. Payment integration can modernize often one of the highest pain points of the health care experience by estimating bills, providing context of charges, and facilitating seamless payment experiences.

## Security and Data Management

The enterprise implementation team will benefit from having a predefined checklist and buy-in from stakeholders. Checklist items include business associate agreements, privacy, risk, legal internal review, IT security review, data management and retention agreements, technical service level agreements, required uptime, support responsibilities and infrastructure, and downtime procedures to minimize business impact. This checklist can help set expectations for the vendor and mitigate downstream miscommunication.

Security and data management are core to all health care, but especially digital solutions. With health care breaches from 2005 to 2019 affecting 249 million individuals, protocols and security measures must be in place to protect patient’s privacy [28]. Each enterprise system is responsible for implementing security compliance, including multifactor authentication logins, secure access and transfer of data, and Health Insurance Portability and Accountability Act compliance [29]. Any partner will need to ensure its capabilities to meet these requirements.

Given all of the data being collected, transmitted, and created, deliberate data management and ownership agreements with the vendor are table stakes for any partnership. As partners, the team should create an understanding of what patient data are being accessed and how and who owns the underlying insights generated. As interoperability policies continue to evolve, patients will maintain ownership of their data with the ability to have access to it and download it, and therefore, vendors should have the capability to support this.

## Scalability

While a digital care pathway may start as a pilot at 1 point in the patient journey in 1 clinical condition, the goal is to eventually service all touchpoints within the care journey across various clinical conditions. When selecting a vendor or platform, it is critical to keep the long-term growth in mind: if our programs are wildly successful, can this platform and vendor meet our enterprise-level needs? There is differential growth in complexity when considering numbers of patients and pathways. Specifically, growth in the number of programs puts pressure on the vendor, while growth in the number of patients engaged on a specific pathway increases requirements from the care team.

In regard to technical robustness, the platform must be dependable both in high-availability uptime and architecture to enable scale. High-availability uptime refers to the percentage of time that a system remains on the internet, as any downtime could be detrimental to the clinical outcomes. A scalable architecture will have technical characteristics that will allow for databases and server needs to grow with the needs of the digital tool without requiring a downstream development lift. In addition, it is important to review the vendor’s service level agreement to understand their expected response times for various degrees of impact.

Beyond the vendor’s technical competencies, the more difficult and differentiating scaling abilities are with the client support and content management teams. As discussed in the *Clinical Content Management* section, these operations manage a highly diverse and rapidly evolving content library, requiring client management of subject matter experts across many service lines. A vendor’s proven ability to manage interfacing across a large organization to navigate complex relationships of multiple clinical partners to effectively grow their content and pathway expertise is critical to providing the best clinical care.

Fundamentally, it is unlikely that a single vendor or solution can address the diverse clinical and practical needs of a large health care enterprise. As a result, it is important to evaluate how interoperable each solution may be with the existing EHR or systems of record.

## Conclusion

Digital patient experience delivers personalized, proactive, and standards-adherent care at scale, placing patient at the center of their care. However, transforming care delivery to support this vision will require enterprise health care systems to use a thoughtful framework encompassing design, organizational implementation, technical requirements, and technology partners to reinvent care delivery. Leveraging a human-centered design methodology, the digital care pathway will ensure the patient’s needs are addressed in navigating complex care of their conditions. On the provider side, redesigning the operational workflows will be critical to allow for facilitating this digital enabled care. When building the underlying technology to run these digital care pathways, delineating the technical requirements of engagement through clinical content management and communication channels, optimization with reporting and analytics, scalability with integration of the EMR, and security are critical to engage with potential partner vendors to deliver an enterprise-scale health system.

As health care looms larger as percentage of gross domestic product, health systems will be continuously guided toward value-based care. This monumental shift from a fee-for-service or a system where hospitals are financially incentivized to fill hospital beds, operating rooms, and ultimately unaccountable to patient outcomes. The shift has slowly started with quality metrics (eg, hospital readmissions) and bundles for surgery care that have downside financial risk for complications. However, a transformation to value-based care in health systems will require a redesign of care delivery, and those able to scale quality practices and patient engagement through digital care pathways will thrive in this transformation. Therefore, while the operational, technological, and organizational costs for this transformation will be large, the old business model of enterprise health systems will not grow in this next chapter of health care.

## Abbreviations

EHR: electronic health record
EMR: electronic medical record
